# Metformindiium dibromide

**DOI:** 10.1107/S2414314626007911

**Published:** 2026-08-11

**Authors:** Aarón Pérez-Benítez, Luis E. Hernández Márquez, Rosa Elena Arroyo-Carmona, Sylvain Bernès

**Affiliations:** a Facultad de Ciencias Químicas, Benemérita Universidad Autónoma de Puebla, Av. San Claudio y 18 Sur, 72570 Puebla, Pue., Mexico; b Instituto de Física Luis Rivera Terrazas, Benemérita Universidad Autónoma de Puebla, Av. San Claudio y 18 Sur, 72570 Puebla, Pue., Mexico; Goethe-Universität Frankfurt, Germany

**Keywords:** crystal structure, metformin, dibromide salt, hydrogen bonds, QTAIM

## Abstract

The crystal structure of the title salt, C_4_H_13_N_5_^2+^·2Br^−^, is reported in space group *P*2_1_/*c*, with *Z* = 4. Unexpectedly, this crystal is not isomorphous to the dichloride analogue, which has been reported in space group *P*2_1_ (*Z* = 2). This originates from the shift of one halide anion in the asymmetric unit, which modifies the network of N—H⋯halide hydrogen bonds. For the here-reported structure, centrosymmetric *R*_4_^2^(8) ring motifs are formed, affording a one-dimensional structure along [100], and the network is further expanded to a three-dimensional structure including larger ring motifs. This report is an example of a routine refinement carried out using now well-established techniques of quantum crystallography.

## Abstract 2

The crystal structure of the dibromide salt of metforminium(2+) in space group *P*2_1_/*c* is compared to that of the dichloride analogue, previously reported in space group *P*2_1_.

## Graphical abstract

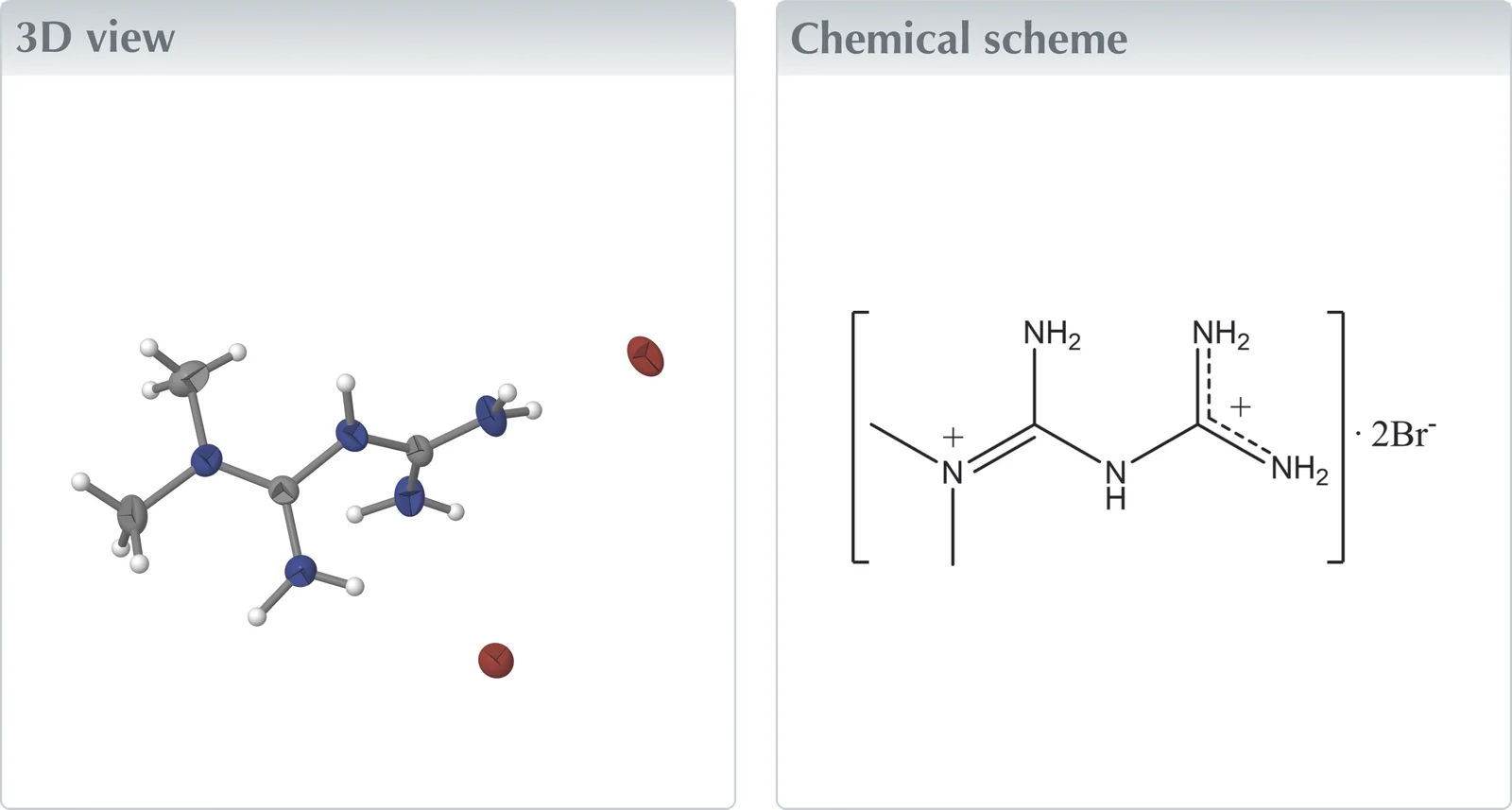

## Structure description

In a recent article, Hill & Boeré (2025 ▸) argue in favour of wider application of quantum crystallography methods in routine chemical crystallography. Nowadays, experimental data quality is enough, even at room temperature, to fit the observed structure factors against non-spherical features of the electronic density map *ρ*(**r**). As a result, refined structures are closer to those expected from neutron diffraction data. Most significantly, accurate positions for H atoms can be obtained, frequently with anisotropic displacement parameters. Such a precision is inherently unachievable in the context of an IAM (Independent Atom Model), since structure factors only include spherical scattering factors. In fact, the default *SFAC* command in *SHELXL* still refers to Cromer–Mann scattering factors for neutral atoms, taken from the 1992 edition of the *Inter­national Tables* (Wilson & Geist, 1992 ▸; Sheldrick, 2015*b* ▸). In opposition, the ‘Hirshfeld Atom Refinement’ approach (HAR; Capelli *et al.*, 2014 ▸; Kleemiss *et al.*, 2021 ▸) uses non-spherical form factors for each atom in the model. The computed map *ρ*(**r**) is then suitable for studying atoms, bonds, and lone pairs, using the concepts devised by Richard Bader in the QTAIM theory (Quantum Theory of Atoms in Mol­ecules; Bader, 2005 ▸). Indeed, some such ‘routine’ structures have already been published in repository journals like *Acta Cryst. C* or *IUCrData* (*e.g*. Jones, 2025 ▸; Samson *et al.*, 2025 ▸). We report here the HAR refinement for a new metforminium(2+) salt.

The title compound, abbreviated H_2_Mf^2+^·2Br^−^, is the dibromide salt of metformin [IUPAC name: [amino­(di­methyl­iminio)meth­yl](di­amino­methyl­idene)ammonium dibromide]. Metformin, Mf, is the first-line oral medication used to lower blood sugar in patients diagnosed with type-2 diabetes, and is sold in form of chlorhydrate, HMf^+^·Cl^−^. On the other hand, the monocation HMf^+^ and dication H_2_Mf^2+^ have been used as counter-ions for vanadium clusters, which are also probed as medication against diabetes (*e.g*. Polito-Lucas *et al.*, 2021 ▸; Chatkon *et al.*, 2022 ▸).

**Figure 1:**
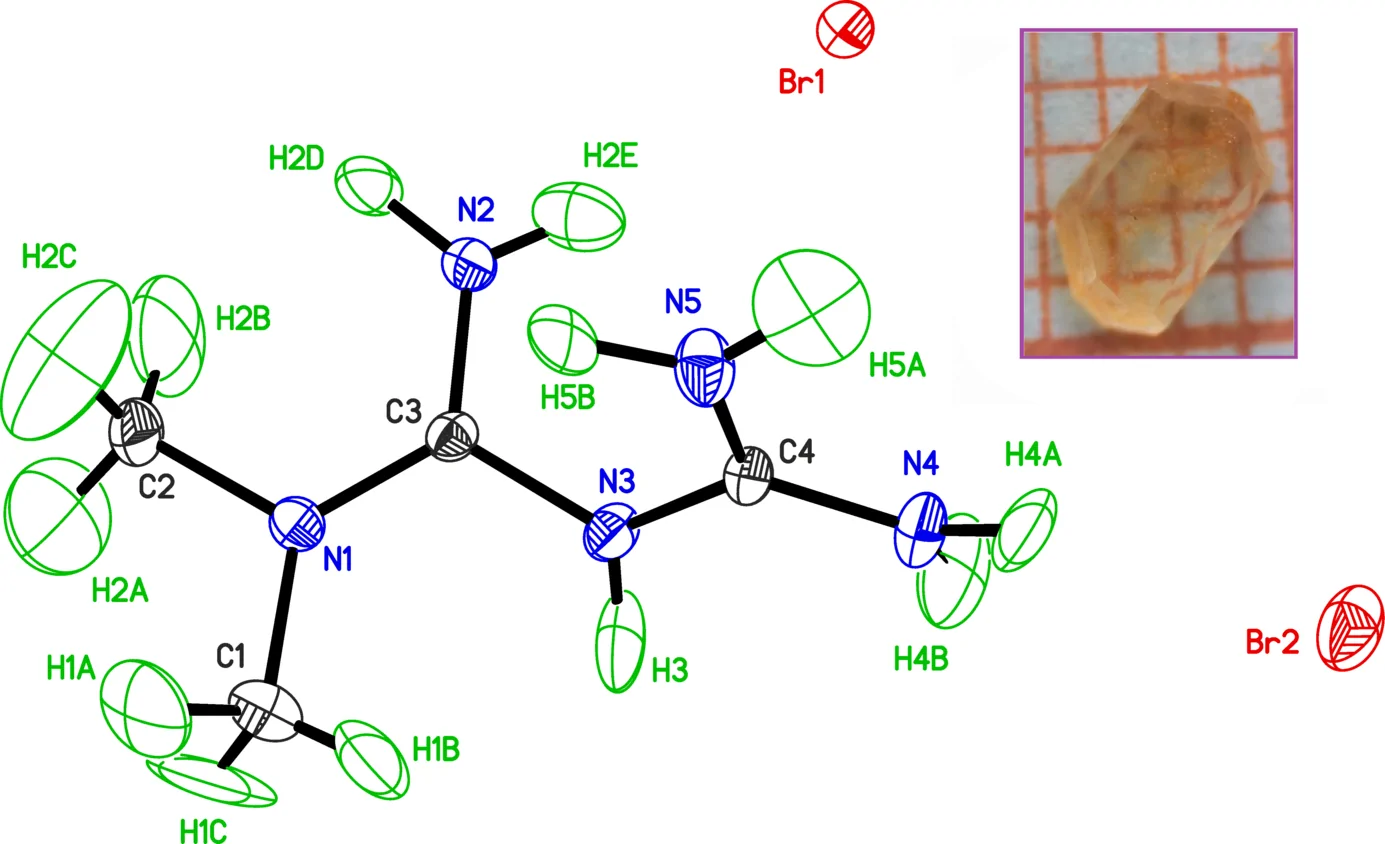
Mol­ecular structure of the title compound, with displacement ellipsoids at 30% probability level. The asymmetric unit and the labelling scheme are those adopted by Xia (2023 ▸) in the dichloride analogue. The inset shows a representative crystal with a maximum dimension of *ca*. 4 mm, obtained by evaporation of an aqueous solution.

**Figure 2:**
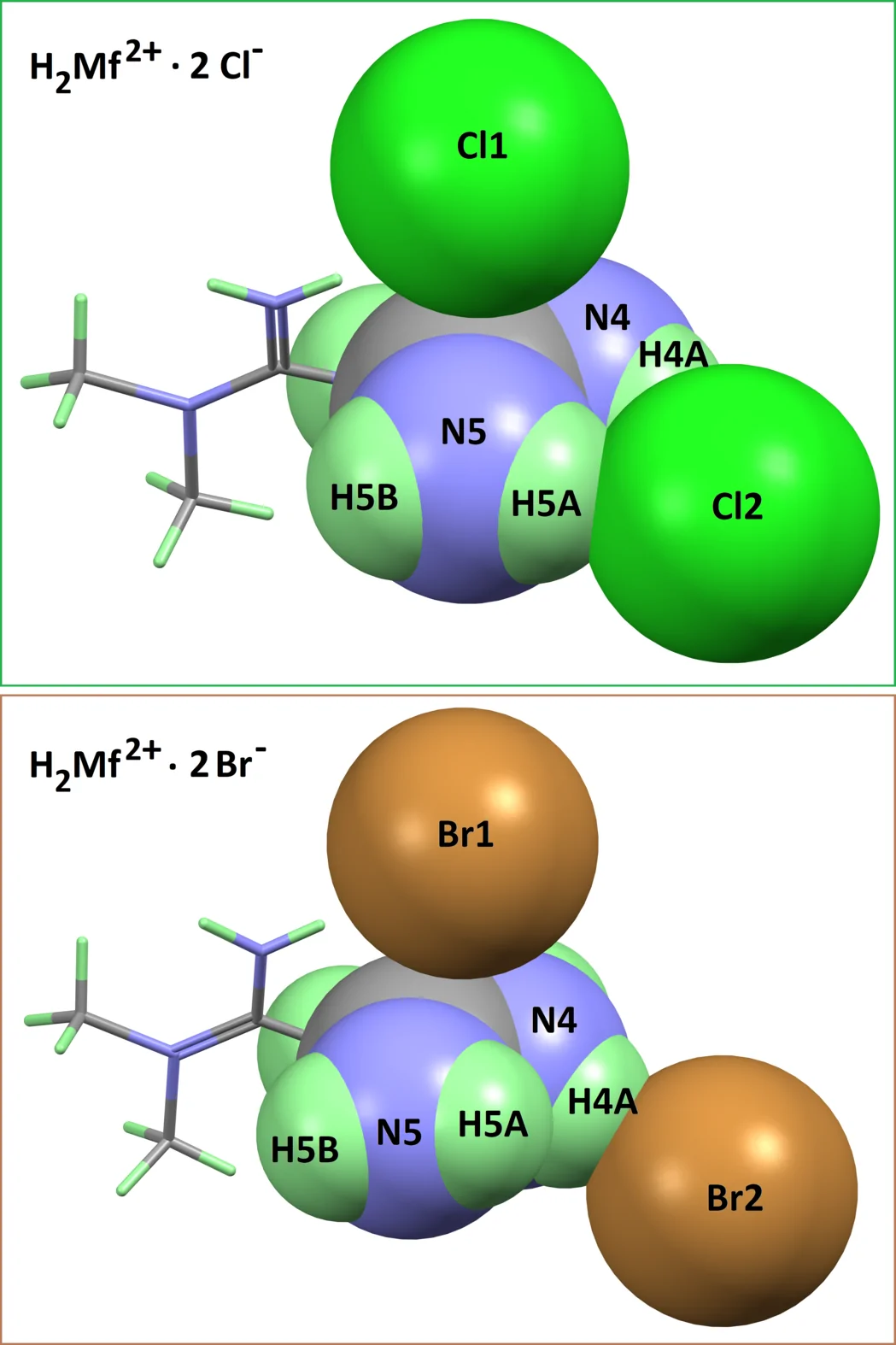
Comparison of the asymmetric units for H_2_Mf^2+^·2Cl^−^ (Xia, 2023 ▸; space group *P*2_1_; top) and H_2_Mf^2+^·2Br^−^ (this work; space group *P*2_1_/*c*; bottom). The guanidine moiety in the cation and halides ions are shown using a spacefill representation (Macrae *et al.*, 2020 ▸).

The here reported crystal structure for H_2_Mf^2+^·2Br^−^ is not isostructural to H_2_Mf^2+^·2Cl^−^ (Xia, 2023 ▸; Hitchings *et al.*, 2025 ▸). The dichloride salt crystallizes in space group *P*2_1_, with *Z* = 2, while the dibromide salt gives crystals in space group *P*2_1_/*c*, with *Z* = 4 (Fig. 1 ▸). Metrics for the dication H_2_Mf^2+^ are however similar in both salts, and its twisted conformation is maintained, regardless of the counter-ion (Table 1 ▸). An overlay between both dications gives an rms deviation of 0.06 Å. The introduction of a *c* glide plane in the case of the dibromide salt should thus result from different positions of the halide ions in the asymmetric units. In turn, these positions should determine different networks of hydrogen bonds. This is indeed the case, as shown in Fig. 2 ▸: the halide ions inter­act directly with the guanidine moiety of H_2_Mf^2+^, through charge-assisted (Cl^−^/Br^−^)⋯(H_2_N)^+^ contacts. Cl1/Br1 ions have the same position in the asymmetric units, and behave as acceptors with H2*E* as donor (Table 2 ▸, entry 1). The key difference relates for Cl2/Br2 position: in the dichloride salt, Cl2 gives a double-acceptor hydrogen bond, with H4*A* and H5*A* as donors (Fig. 2 ▸, top). In the case of the dibromide salt, only one hydrogen bond is formed, N4—H4*A*⋯Br2 (Fig. 2 ▸, bottom; Table 2 ▸, entry 2).

**Figure 3:**
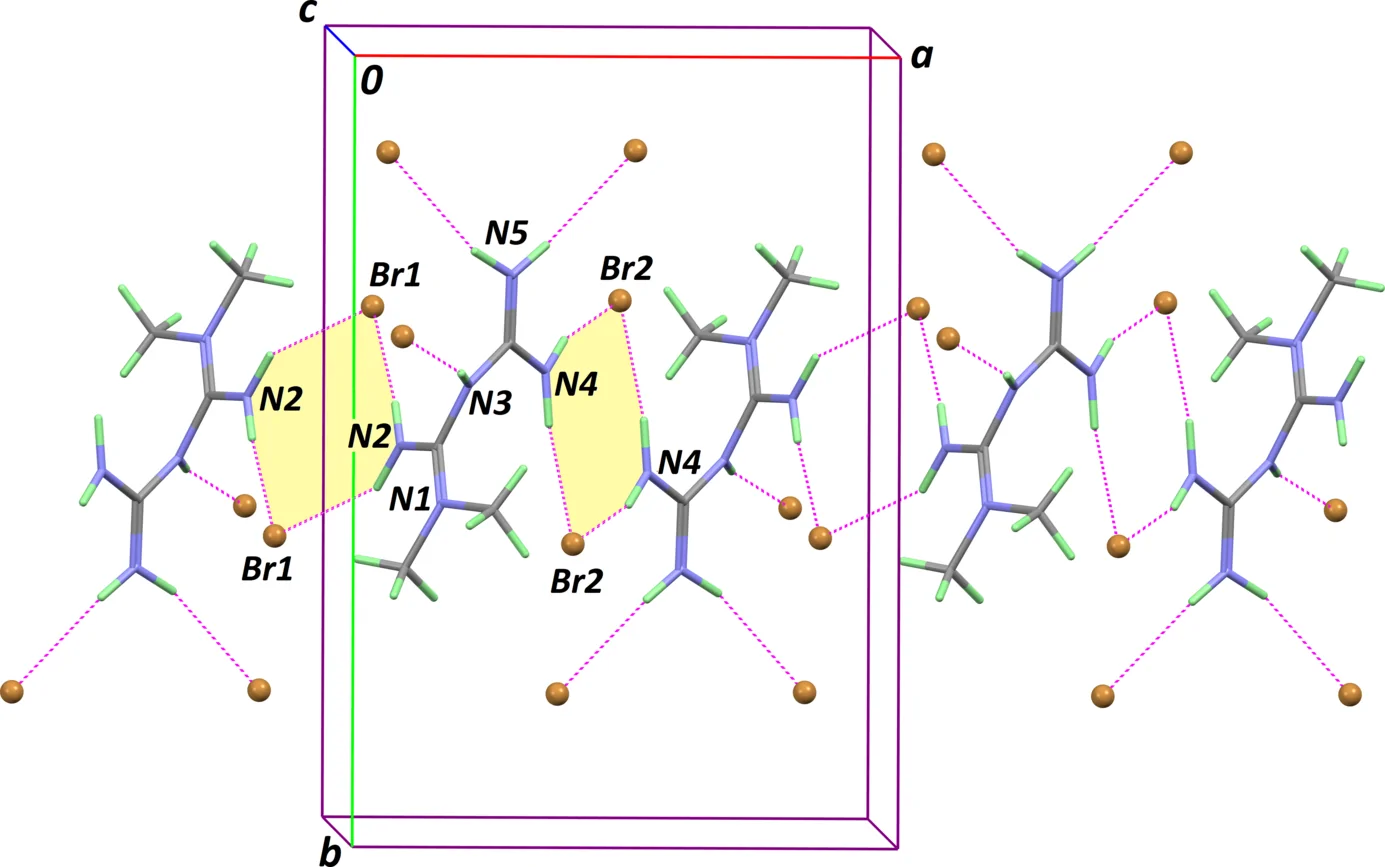
Part of the crystal structure of the title compound, emphasizing 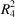
(8) rings building the one-dimensional framework (yellow areas). Br atom inter­actions with N3 and N5 afford a three-dimensional supra­molecular structure.

This difference for the position of one anion is reflected in different supra­molecular features. The smallest ring motifs found in H_2_Mf^2+^·2Br^−^ have graph set 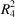
(8) and are centrosymmetric. Cations are connected through these rings, forming a one-dimensional network oriented along [100]. The remaining amine groups, N3 and N5, give other efficient N—H⋯Br inter­actions, to build a complex three-dimensional framework (Fig. 3 ▸). A different image is obtained in the case of the dichloride salt: the bifurcated hydrogen bond including Cl2 affords an 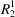
(6) ring motif, which is not present in the dibromide salt. The complete crystal structure is essentially diperiodic, and expands to a three-dimensional framework, documented in the supplementary information file available from the *CrystEngComm* article (Hitchings *et al.*, 2025 ▸; see Table S5 and Figure S3).

## Synthesis and crystallization

The title compound was synthesized by reacting at room temperature 3.4 g (20.5 mmol) of metforminium chloride dissolved in 15 ml of distilled water, with 6 ml of 48% HBr (53.0 mmol). After 15 min. of magnetic stirring, the solvent was evaporated at ambient conditions for about three weeks, to yield large polyhedric colourless single crystals (Fig. 1 ▸, inset).

## Refinement

Crystal data, data collection and structure refinement details are summarized in Table 3 ▸. The structure was first refined with *SHELXL* (Sheldrick, 2015*b* ▸) and then with *OLEX2.refine* using the GUI *OLEX2* (Bourhis *et al.*, 2015 ▸; Dolomanov *et al.*, 2009 ▸). Further refinements were carried out using *NoSpherA2* in the same GUI (Kleemiss *et al.*, 2021 ▸) and *ORCA* for DFT computations (Neese, 2022 ▸). The last cycles for refining non-spherical scattering factors were based on a tetra­meric model, [C_4_H_13_N_5_Br_2_]_4_, in order to take in account non-covalent bonds, using the PBE0 functional in conjunction with the def2-TZVPD basis set, including the DKH2 correction for relativistic effects. Coordinates and anisotropic displacement parameters were refined for all atoms, without restraints nor constraints. A batch of 12 cycles of refinement for this 96-atom model including 568 electrons is completed in a few hours on an unsophisticated desktop PC (i7–6700 CPU @3.40 GHz, 8 threads, 8 Gb RAM).

## Supplementary Material

Crystal structure: contains datablock(s) I. DOI: 10.1107/S2414314626007911/bt4203sup1.cif

Structure factors: contains datablock(s) I. DOI: 10.1107/S2414314626007911/bt4203Isup2.hkl

Supporting information file. DOI: 10.1107/S2414314626007911/bt4203Isup3.cml

CCDC reference: 2578518

Additional supporting information:  crystallographic information; 3D view; checkCIF report

## Figures and Tables

**Table 1:** Bond lengths (Å) and valence angles (°) observed in dication H_2_Mf^2+^ for the dichloride (Xia, 2023 ▸) and dibromide salts

Parameter	H_2_Mf^2+^·2Cl^−*a*^	H_2_Mf^2+^·2Br^−^/*SHELXL*^*b*^	H_2_Mf^2+^·2Br^−^/*NoSpherA2*^*c*^
Bonds			
N1—C1	1.473 (4)	1.463 (3)	1.455 (3)
N1—C2	1.469 (4)	1.462 (4)	1.459 (4)
N1—C3	1.335 (4)	1.304 (3)	1.309 (3)
N2—C3	1.321 (4)	1.315 (3)	1.315 (3)
N3—C3	1.386 (3)	1.392 (3)	1.392 (3)
N3—C4	1.378 (3)	1.362 (3)	1.362 (3)
N4—C4	1.325 (4)	1.306 (4)	1.309 (4)
N5—C4	1.310 (4)	1.304 (4)	1.311 (3)
Angles			
C1—N1—C2	115.7 (3)	115.1 (3)	115.2 (3)
C1—N1—C3	122.9 (2)	123.2 (2)	123.3 (3)
C2—N1—C3	120.0 (3)	120.9 (2)	120.7 (2)
N1—C3—N2	123.3 (2)	123.6 (2)	123.5 (2)
N1—C3—N3	118.2 (2)	119.5 (2)	119.2 (2)
N2—C3—N3	118.3 (3)	116.8 (2)	117.3 (2)
C3—N3—C4	125.0 (2)	124.8 (2)	124.4 (2)
N3—C4—N4	118.1 (3)	118.0 (3)	117.9 (3)
N3—C4—N5	120.4 (3)	119.5 (2)	119.6 (2)
N4—C4—N5	121.6 (3)	122.5 (3)	122.4 (3)
Torsion angles			
N1—C3—N3—C4	−139.2 (3)	−133.1 (3)	−133.4 (2)
N2—C3—N3—C4	45.6 (4)	49.7 (4)	49.6 (3)

Notes: (*a*) Xia (2023 ▸); (*b*) *SHELXL* refinement: isotropic H atoms; free coordinates and displacement parameters for all atoms (152 parameters, *R*_1_ = 3.14% for observed reflections, *wR*_2_ = 7.84% for all reflections); (*c*) refinement details in Table 3 ▸.

**Table 2:** Hydrogen-bond geometry (Å, °)

*D*—H⋯*A*	*D*—H	H⋯*A*	*D*⋯*A*	*D*—H⋯*A*
N2—H2*E*⋯Br1	0.89 (5)	2.46 (4)	3.343 (3)	168 (3)
N4—H4*A*⋯Br2	0.91 (5)	2.48 (4)	3.355 (3)	163 (3)
N2—H2*D*⋯Br1^i^	0.99 (4)	2.45 (4)	3.341 (3)	149 (3)
N3—H3⋯Br1^ii^	0.94 (4)	2.42 (4)	3.321 (2)	160 (4)
N4—H4*B*⋯Br1^iii^	1.01 (5)	2.48 (5)	3.350 (3)	144 (4)
N5—H5*A*⋯Br2^iv^	0.90 (5)	2.49 (5)	3.346 (3)	157 (5)
N5—H5*B*⋯Br2^v^	0.99 (4)	2.34 (4)	3.322 (3)	172 (3)

Symmetry codes: (i) 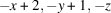; (ii) 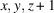; (iii) 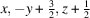; (iv) 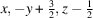; (v) 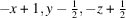.

**Table 3:** Experimental details

Crystal data	
Chemical formula	C_4_H_13_N_5_^2+^·2Br^−^
*M*_r_	290.99
Crystal system, space group	Monoclinic, *P*2_1_/*c*
Temperature (K)	295
*a*, *b*, *c* (Å)	10.3335 (3), 15.0443 (4), 6.4977 (2)
β (°)	95.427 (2)
*V* (Å^3^)	1005.61 (5)
*Z*	4
Radiation type	Ag *K*α, λ = 0.56083 Å
μ (mm^−1^)	4.29
Crystal size (mm)	0.29 × 0.20 × 0.07

Data collection	
Diffractometer	Stoe Stadivari
Absorption correction	Multi-scan (*LANA*; Folkers-Karlsson *et al.*, 2026 ▸)
*T*_min_, *T*_max_	0.288, 0.741
No. of measured, independent and observed [*I* > 2σ(*I*)] reflections	31050, 3212, 2465
*R*_int_	0.031
(sin θ/λ)_max_ (Å^−1^)	0.725

Refinement	
*R*[*F*^2^ > 2σ(*F*^2^)], *wR*(*F*^2^), *S*	0.030, 0.070, 1.04
No. of reflections	3212
No. of parameters	217
H-atom treatment	All H-atom parameters refined
Δρ_max_, Δρ_min_ (e Å^−3^)	0.85, −0.57

Computer programs: *X-AREAPilatus3SV* (Stoe, 2025 ▸), *X-AREARecipe* (Folkers-Karlsson *et al.*, 2026 ▸), *X-AREAIntegrate3D* and *LANA* (Folkers-Karlsson *et al.*, 2026 ▸), *SHELXT2018/2* (Sheldrick, 2015*a* ▸), *OLEX2.refine* (Bourhis *et al.*, 2015 ▸), *XP* in *SHELXTL-Plus* (Sheldrick, 2008 ▸), *Mercury* (Macrae *et al.*, 2020 ▸) and *publCIF* (Westrip, 2010 ▸).

## full crystallographic data

### [Amino(dimethyliminio)methyl](diaminomethylidene)ammonium dibromide Crystal data

**Table:**

C_4_H_13_N_5_^2+^·2Br^−^	*F*(000) = 569.890
*M**_r_* = 290.99	*D*_x_ = 1.922 Mg m^−^^3^
Monoclinic, *P*2_1_/*c*	Ag *K*α radiation, λ = 0.56083 Å
*a* = 10.3335 (3) Å	Cell parameters from 22061 reflections
*b* = 15.0443 (4) Å	θ = 2.7–29.9°
*c* = 6.4977 (2) Å	µ = 4.29 mm^−^^1^
β = 95.427 (2)°	*T* = 295 K
*V* = 1005.61 (5) Å^3^	Plate, colourless
*Z* = 4	0.29 × 0.2 × 0.07 mm

### [Amino(dimethyliminio)methyl](diaminomethylidene)ammonium dibromide Data collection

**Table:**

Stoe Stadivari diffractometer	3212 independent reflections
Radiation source: Sealed X-ray tube, Axo Astix-f Microfocus source	2465 reflections with *I* > 2σ(*I*)
Graded multilayer mirror monochromator	*R*_int_ = 0.031
Detector resolution: 5.81 pixels mm^-1^	θ_max_ = 24.0°, θ_min_ = 2.7°
ω scans	*h* = −14→14
Absorption correction: multi-scan (*LANA*;Folkers-Karlsson *et al*., 2026)	*k* = −21→21
*T*_min_ = 0.288, *T*_max_ = 0.741	*l* = −9→9
31050 measured reflections	

### [Amino(dimethyliminio)methyl](diaminomethylidene)ammonium dibromide Refinement

**Table:**

Refinement on *F*^2^	0 constraints
Least-squares matrix: full	Primary atom site location: dual
*R*[*F*^2^ > 2σ(*F*^2^)] = 0.030	Secondary atom site location: difference Fourier map
*wR*(*F*^2^) = 0.070	All H-atom parameters refined
*S* = 1.04	*w* = 1/[σ^2^(*F*_o_^2^) + (0.0267*P*)^2^ + 1.1908*P*] where *P* = (*F*_o_^2^ + 2*F*_c_^2^)/3
3212 reflections	(Δ/σ)_max_ = 0.001
217 parameters	Δρ_max_ = 0.85 e Å^−^^3^
0 restraints	Δρ_min_ = −0.57 e Å^−^^3^

### [Amino(dimethyliminio)methyl](diaminomethylidene)ammonium dibromide Fractional atomic coordinates and isotropic or equivalent isotropic displacement parameters (Å^2^)

**Table:**

	*x*	*y*	*z*	*U*_iso_*/*U*_eq_	
Br1	0.90769 (3)	0.642983 (18)	−0.06038 (4)	0.03418 (8)	
Br2	0.44558 (3)	0.85428 (2)	0.28006 (5)	0.04693 (9)	
N1	0.81449 (19)	0.41933 (14)	0.5843 (3)	0.0288 (4)	
N2	0.8743 (3)	0.48014 (19)	0.2806 (4)	0.0341 (5)	
H2D	0.916 (4)	0.426 (3)	0.231 (5)	0.048 (10)	
H2E	0.881 (4)	0.529 (3)	0.205 (6)	0.061 (12)	
N3	0.7614 (2)	0.56611 (16)	0.4982 (3)	0.0307 (5)	
H3	0.783 (4)	0.585 (3)	0.635 (7)	0.065 (12)	
N4	0.6770 (3)	0.70012 (18)	0.3858 (5)	0.0377 (5)	
H4A	0.612 (4)	0.736 (3)	0.330 (7)	0.058 (12)	
H4B	0.749 (4)	0.735 (3)	0.465 (8)	0.079 (15)	
N5	0.6060 (3)	0.5722 (2)	0.2185 (4)	0.0354 (5)	
H5A	0.568 (3)	0.608 (4)	0.118 (8)	0.078 (15)	
H5B	0.600 (4)	0.506 (3)	0.215 (5)	0.046 (10)	
C1	0.7302 (4)	0.4179 (3)	0.7514 (6)	0.0441 (7)	
H1A	0.671 (5)	0.360 (3)	0.730 (7)	0.080 (15)	
H1B	0.675 (5)	0.468 (3)	0.748 (7)	0.102 (18)	
H1C	0.780 (5)	0.402 (4)	0.878 (6)	0.12 (2)	
C2	0.8806 (4)	0.3358 (2)	0.5493 (6)	0.0424 (7)	
H2A	0.910 (6)	0.306 (4)	0.683 (8)	0.13 (2)	
H2B	0.964 (7)	0.343 (3)	0.475 (12)	0.14 (3)	
H2C	0.824 (5)	0.295 (4)	0.451 (12)	0.15 (3)	
C3	0.8168 (2)	0.48498 (16)	0.4524 (3)	0.0241 (4)	
C4	0.6799 (2)	0.61371 (16)	0.3628 (4)	0.0257 (4)	

### [Amino(dimethyliminio)methyl](diaminomethylidene)ammonium dibromide Atomic displacement parameters (Å^2^)

**Table:**

	*U*^11^	*U*^22^	*U*^33^	*U*^12^	*U*^13^	*U*^23^
Br1	0.03867 (13)	0.03190 (14)	0.03194 (12)	0.00332 (10)	0.00313 (9)	−0.00091 (10)
Br2	0.05447 (18)	0.04031 (17)	0.04234 (15)	0.01947 (13)	−0.01468 (12)	−0.00801 (12)
N1	0.0322 (10)	0.0297 (11)	0.0238 (9)	0.0024 (8)	−0.0007 (7)	0.0022 (8)
N2	0.0430 (13)	0.0310 (13)	0.0294 (11)	0.0084 (11)	0.0104 (10)	0.0042 (10)
H2D	0.06 (3)	0.05 (3)	0.04 (2)	0.03 (2)	0.017 (19)	0.01 (2)
H2E	0.06 (3)	0.08 (3)	0.05 (3)	−0.02 (2)	0.01 (2)	0.00 (2)
N3	0.0358 (11)	0.0285 (11)	0.0262 (10)	0.0087 (9)	−0.0055 (8)	−0.0039 (9)
H3	0.08 (3)	0.03 (2)	0.09 (3)	0.01 (2)	−0.01 (3)	0.01 (2)
N4	0.0376 (13)	0.0237 (12)	0.0518 (16)	0.0065 (10)	0.0042 (11)	0.0007 (11)
H4A	0.08 (3)	0.02 (2)	0.08 (3)	0.01 (2)	0.03 (2)	−0.01 (2)
H4B	0.06 (3)	0.06 (3)	0.11 (4)	0.03 (3)	−0.03 (3)	0.00 (3)
N5	0.0348 (12)	0.0298 (13)	0.0391 (13)	0.0012 (10)	−0.0091 (10)	0.0038 (11)
H5A	0.02 (2)	0.11 (4)	0.11 (4)	0.00 (2)	0.02 (2)	0.01 (3)
H5B	0.07 (3)	0.04 (2)	0.019 (18)	0.00 (2)	−0.016 (17)	0.007 (17)
C1	0.0488 (18)	0.052 (2)	0.0331 (15)	−0.0020 (16)	0.0106 (13)	0.0068 (15)
H1A	0.09 (3)	0.07 (3)	0.08 (3)	−0.05 (3)	0.02 (3)	0.00 (2)
H1B	0.16 (4)	0.07 (3)	0.09 (3)	0.05 (3)	0.08 (3)	0.05 (3)
H1C	0.15 (5)	0.18 (6)	0.04 (2)	0.03 (4)	0.06 (3)	0.03 (3)
C2	0.057 (2)	0.0293 (15)	0.0398 (16)	0.0100 (14)	0.0006 (14)	0.0062 (12)
H2A	0.17 (6)	0.12 (5)	0.09 (4)	0.04 (4)	−0.04 (4)	0.03 (4)
H2B	0.17 (6)	0.07 (4)	0.18 (6)	0.06 (4)	0.07 (6)	0.05 (4)
H2C	0.06 (3)	0.12 (5)	0.25 (7)	−0.01 (3)	−0.04 (4)	−0.09 (5)
C3	0.0247 (10)	0.0249 (11)	0.0219 (9)	0.0021 (8)	−0.0015 (8)	−0.0024 (8)
C4	0.0262 (10)	0.0215 (10)	0.0293 (10)	0.0036 (9)	0.0020 (8)	0.0008 (9)

### [Amino(dimethyliminio)methyl](diaminomethylidene)ammonium dibromide Geometric parameters (Å, º)

**Table:**

N1—C1	1.455 (3)	N4—C4	1.309 (4)
N1—C2	1.459 (4)	N5—H5A	0.90 (5)
N1—C3	1.309 (3)	N5—H5B	0.99 (4)
N2—H2D	0.99 (4)	N5—C4	1.311 (3)
N2—H2E	0.89 (5)	C1—H1A	1.06 (4)
N2—C3	1.315 (3)	C1—H1B	0.95 (4)
N3—H3	0.94 (4)	C1—H1C	0.96 (5)
N3—C3	1.392 (3)	C2—H2A	1.00 (5)
N3—C4	1.362 (3)	C2—H2B	1.04 (6)
N4—H4A	0.91 (5)	C2—H2C	1.02 (5)
N4—H4B	1.01 (5)		
			
C2—N1—C1	115.2 (3)	H1B—C1—H1A	108 (4)
C3—N1—C1	123.3 (3)	H1C—C1—N1	109 (3)
C3—N1—C2	120.7 (2)	H1C—C1—H1A	99 (4)
H2E—N2—H2D	116 (3)	H1C—C1—H1B	120 (4)
C3—N2—H2D	125 (2)	H2A—C2—N1	111 (3)
C3—N2—H2E	119 (3)	H2B—C2—N1	114 (3)
C3—N3—H3	114 (2)	H2B—C2—H2A	105 (5)
C4—N3—H3	122 (2)	H2C—C2—N1	111 (3)
C4—N3—C3	124.4 (2)	H2C—C2—H2A	113 (5)
H4B—N4—H4A	113 (4)	H2C—C2—H2B	103 (5)
C4—N4—H4A	125 (3)	N2—C3—N1	123.5 (2)
C4—N4—H4B	123 (2)	N3—C3—N1	119.2 (2)
H5B—N5—H5A	123 (4)	N3—C3—N2	117.3 (2)
C4—N5—H5A	115 (3)	N4—C4—N3	117.9 (3)
C4—N5—H5B	121.7 (19)	N5—C4—N3	119.6 (2)
H1A—C1—N1	107 (2)	N5—C4—N4	122.4 (3)
H1B—C1—N1	112 (2)		
			
N1—C3—N3—C4	−133.4 (2)	N2—C3—N3—C4	49.6 (3)

### [Amino(dimethyliminio)methyl](diaminomethylidene)ammonium dibromide Hydrogen-bond geometry (Å, º)

**Table:**

*D*—H···*A*	*D*—H	H···*A*	*D*···*A*	*D*—H···*A*
N2—H2*E*···Br1	0.89 (5)	2.46 (4)	3.343 (3)	168 (3)
N4—H4*A*···Br2	0.91 (5)	2.48 (4)	3.355 (3)	163 (3)
N2—H2*D*···Br1^i^	0.99 (4)	2.45 (4)	3.341 (3)	149 (3)
N3—H3···Br1^ii^	0.94 (4)	2.42 (4)	3.321 (2)	160 (4)
N4—H4*A*···N4^iii^	0.91 (5)	3.18 (4)	3.579 (2)	109 (3)
N4—H4*B*···Br1^iv^	1.01 (5)	2.48 (5)	3.350 (3)	144 (4)
N4—H4*B*···N4^iv^	1.01 (5)	3.06 (5)	3.579 (2)	113 (3)
N5—H5*A*···Br2^iii^	0.90 (5)	2.49 (5)	3.346 (3)	157 (5)
N5—H5*B*···Br2^v^	0.99 (4)	2.34 (4)	3.322 (3)	172 (3)
C1—H1*A*···N5^vi^	1.06 (4)	3.08 (5)	3.501 (5)	104 (3)
C1—H1*B*···N3	0.95 (4)	2.43 (4)	2.808 (5)	104 (3)
C1—H1*B*···N5^vi^	0.95 (4)	2.99 (5)	3.501 (5)	115 (4)
C2—H2*A*···Br1^vii^	1.00 (5)	3.24 (6)	3.630 (3)	105 (4)

Symmetry codes: (i) −*x*+2, −*y*+1, −*z*; (ii) *x*, *y*, *z*+1; (iii) *x*, −*y*+3/2, *z*−1/2; (iv) *x*, −*y*+3/2, *z*+1/2; (v) −*x*+1, *y*−1/2, −*z*+1/2; (vi) −*x*+1, −*y*+1, −*z*+1; (vii) −*x*+2, *y*−1/2, −*z*+1/2.
